# Activated protein C ameliorates coagulopathy but does not influence outcome in lethal H1N1 influenza: a controlled laboratory study

**DOI:** 10.1186/cc8964

**Published:** 2010-04-14

**Authors:** Marcel Schouten, Koenraad F van der Sluijs, Bruce Gerlitz, Brian W Grinnell, Joris JTH Roelofs, Marcel M Levi, Cornelis van 't Veer, Tom van der Poll

**Affiliations:** 1 Center for Experimental and Molecular Medicine (CEMM), Academic Medical Center, University of Amsterdam, Meibergdreef 9, Room G2-130, 1105 AZ, Amsterdam, The Netherlands; 2 Center for Infection and Immunity Amsterdam (CINIMA), Academic Medical Center, University of Amsterdam, Meibergdreef 9, Room G2-130, 1105 AZ, Amsterdam, The Netherlands; 3 Laboratory of Experimental Immunology, Academic Medical Center, University of Amsterdam, Meibergdreef 9, Room G2-130, 1105 AZ, Amsterdam, The Netherlands; 4 Department of Pulmonology; Academic Medical Center, Academic Medical Center, University of Amsterdam, Meibergdreef 9, Room G2-130, 1105 AZ, Amsterdam, The Netherlands; 5 Biotechnology Discovery Research, Lilly Research Laboratories; Lilly Corporate Center, Indianapolis, Indiana, IN 46285-0444, USA; 6 Department of Pathology, Academic Medical Center, University of Amsterdam, Meibergdreef 9, Room G2-130, 1105 AZ, Amsterdam, The Netherlands; 7 Department of Internal Medicine, Academic Medical Center, University of Amsterdam, Meibergdreef 9, Room G2-130, 1105 AZ, Amsterdam, The Netherlands

## Abstract

**Introduction:**

Influenza accounts for 5 to 10% of community-acquired pneumonias and is a major cause of mortality. Sterile and bacterial lung injuries are associated with procoagulant and inflammatory derangements in the lungs. Activated protein C (APC) is an anticoagulant with anti-inflammatory properties that exert beneficial effects in models of lung injury. We determined the impact of lethal influenza A (H1N1) infection on systemic and pulmonary coagulation and inflammation, and the effect of recombinant mouse (rm-) APC hereon.

**Methods:**

Male C57BL/6 mice were intranasally infected with a lethal dose of a mouse adapted influenza A (H1N1) strain. Treatment with rm-APC (125 μg intraperitoneally every eight hours for a maximum of three days) or vehicle was initiated 24 hours after infection. Mice were euthanized 48 or 96 hours after infection, or observed for up to nine days.

**Results:**

Lethal H1N1 influenza resulted in systemic and pulmonary activation of coagulation, as reflected by elevated plasma and lung levels of thrombin-antithrombin complexes and fibrin degradation products. These procoagulant changes were accompanied by inhibition of the fibrinolytic response due to enhanced release of plasminogen activator inhibitor type-1. Rm-APC strongly inhibited coagulation activation in both plasma and lungs, and partially reversed the inhibition of fibrinolysis. Rm-APC temporarily reduced pulmonary viral loads, but did not impact on lung inflammation or survival.

**Conclusions:**

Lethal influenza induces procoagulant and antifibrinolytic changes in the lung which can be partially prevented by rm-APC treatment.

## Introduction

Influenza A infection is a major cause of morbidity and mortality: Seasonal influenza A infection causes over 200,000 hospitalizations and approximately 41,000 deaths in the United States annually, being the seventh leading cause of mortality [1]. Besides its regular seasonal character, influenza A, due to the introduction and adaptation of novel hemagglutinin subtypes from other mammals or birds resulting in antigenic shifts, has the potential to cause pandemics, as the pandemics in 1918, 1957 and 1968 have shown [2]. Currently, a novel influenza A (H1N1) strain from swine origin has evolved to a pandemic, now worldwide causing major concern for the near future [3]. Although the greatest proportion of mortality caused by influenza A infection is due to secondary bacterial pneumonia and cardiovascular complications, influenza itself is also an important cause of community-acquired pneumonia (CAP), causing 5 to 10% of CAP-cases [4-7]. As such, influenza is a major concern for pulmonologists and intensive care physicians [8].

Severe infection and inflammation have been closely linked to activation of coagulation and downregulation of anticoagulant mechanisms and fibrinolysis [9]. In bacterial pneumonia, pulmonary activation of coagulation as well as downregulation of the anticoagulant protein C (PC) pathway and fibrinolysis have been demonstrated [10-12]. Beside anticoagulant properties, activated (A)PC has been shown to have profibrinolytic, anti-inflammatory, anti-apoptotic and other cytoprotective properties [13]. Downregulation of the PC pathway has been correlated to disease severity and mortality in severe bacterial pneumonia and sepsis [14,15] and continuous intravenous administration of recombinant human (rh-) APC for four days (Human Activated Protein C Worldwide Evaluation in Severe Sepsis (PROWESS) trial) has been shown not only to downregulate activation of coagulation, but also to reduce inflammation and improve survival in patients with severe sepsis [16]. The benefical effect of rh-APC in this trial seemed especially prominent in patients with severe sepsis due to pneumonia [17]. While much research has been done on coagulation activation during severe bacterial infection, data on coagulation activation in viral infection like influenza are sparse. Evidence that influenza can be associated with coagulation activation comes from a clinical study in pediatric patients hospitalized for severe influenza [18] and from a recent study showing elevated plasma levels of thrombin-antithrombin complexes (TATc) in mice infected with a non-lethal dose of influenza A [19]. Interestingly, and as mentioned above, many elderly patients with influenza infections suffer from cardiovascular complications.

At present it is unknown whether APC can influence the procoagulant and inflammatory response to lethal influenza A infection. Therefore, in the present study we sought to establish the effect of recombinant mouse (rm)-APC treatment on local and systemic activation of coagulation and fibrinolysis during lethal H1N1 influenza A in mice and moreover determined the effect of rm-APC on lung inflammation, pulmonary viral loads and survival. We here show, that lethal H1N1 influenza A infection is associated with both pulmonary and systemic activation of coagulation and inhibition of fibrinolysis. Moreover, we show that rm-APC treatment, started 24 hours after the onset of infection, partially prevents these hemostatic derangements, but does not impact on lung inflammation or survival.

## Materials and methods

### Animals

Male C57BL/6 mice were purchased from Charles River (Maastricht, the Netherlands) and maintained in the animal facility of the Academic Medical Center (University of Amsterdam) according to national guidelines with free access to food and water. Ten-week-old mice were used in experiments. All experiments were approved by the Institutional Animal Care and Use Committee of the Academic Medical Center.

### Experimental infection and treatment

Influenza infection was induced by intranasal instillation of a lethal dose (28,000 copies) of influenza A/PR/8/34 (H1N1, ATCC no. VR-95; Rockville, MD, USA), as described [20,21]. This infectious dose was chosen based on a previous study from our laboratory showing that it caused lethality in C57BL/6 mice which could be delayed by eliminating signalling via the proinflammatory receptor for advanced glycation end products (RAGE) [20]. Recombinant murine (rm-) APC and buffer control were generated by Eli Lilly & Co (Indianapolis, IN, USA) as described [22]. Rm-APC (2 mg/ml) was diluted in sterile pyrogen-free saline to a concentration of 625 μg/ml. The buffer control was diluted likewise. Uninfected mice were euthanized before or at one, four or eight hours (n = 4 per time point) after a single intraperitoneal injection of 125 μg of rm-APC (200 μl) to determine plasma APC-levels. In infection experiments, from 24 hours after infection on, mice were treated every eight hours for a maximum of three days with 125 μg of rm-APC or buffer. Sample harvesting and processing, and determination of viral copies were done as described (n = 8 per group at each time point) [20,21].

### Assays

M-APC levels were measured by an enzyme capture assay [23]. TATc (Behringwerke AG, Marburg, Germany), fibrin degradation products (FDP) [24], plasminogen activator inhibitor type-1 antigen (PAI-1) [25], myeloperoxidase (MPO; HyCult Biotechnology, Uden, the Netherlands), interleukin (IL)-1β, keratinocyte-derived chemokine (KC) and macrophage inflammatory protein (MIP)-2 (all R&D Systems, Minneapolis, MN, USA) were measured by ELISA. Plasminogen activator activity (PAA) was determined by an amidolytic assay [26]. Tumor necrosis factor (TNF)-α, IL-6, IL-12p70, IL-10 and interferon (IFN)-γ were measured by cytometric bead array (CBA) multiplex assay (BD Biosciences, San Jose, CA, USA).

### Histology and immunohistochemistry

Paraffin lung sections were stained with haematoxylin and eosin or fluorescein isothiocyanate-labeled anti-mouse Ly-6G mAb (Pharmingen, San Diego, CA, USA) as described [27]. To score lung inflammation, the lung surface was analyzed with respect to the following parameters: bronchitis, interstitial inflammation, oedema, endothelialitis, pleuritis and thrombus formation. Each parameter was graded on a scale of 0 to 4 (0: absent, 1: mild, 2: moderate, 3: severe, 4: very severe). The total histopathological score was expressed as the sum of the scores for the different parameters, the maximum being 24. Ly-6G stained slides were photographed with a microscope equipped with a digital camera (Leica CTR500, Leica Microsystems, Wetzlar, Germany). Stained areas were analysed with Image Pro Plus (Media Cybernetics, Bethesda, MD, USA) and expressed as percentage of the surface area. The average of 10 pictures was used for analysis.

### Statistical analysis

Data are expressed as box-and-whisker diagrams (depicting the smallest observation, lower quartile, median, upper quartile and largest observation), medians with interquartile ranges or as survival curves. Differences between groups were determined with Kruskal-Wallis, Mann-Whitney *U *test or log rank test. Analyses were performed using GraphPad Prism version 4.0 (GraphPad Software, San Diego, CA, USA). *P*-values less than 0.05 were considered statistically significant.

## Results

### Plasma m-APC levels after single dose administration of rm-APC

To determine plasma levels of m-APC after single dose administration of rm-APC, uninfected mice were injected intraperitoneally with 125 μg of rm-APC (200 μl) and sacrificed after one, four or eight hours. Plasma levels of rm-APC after single dose administration were 154 (interquartile range 76 to 250), 122 (96 to 131) and 33 (27 to 40) ng/ml after one, four and eight hours, respectively.

### Activation of coagulation and downregulation of fibrinolysis

**Figure 1:**
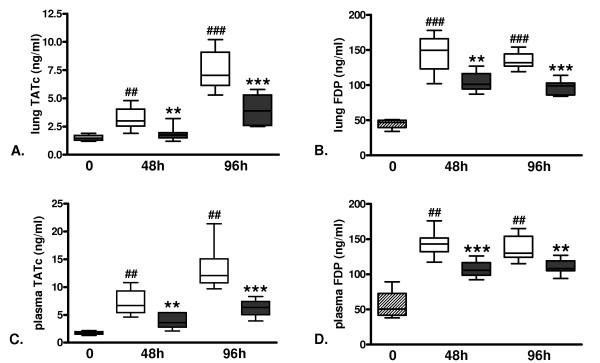
**Activation of coagulation in lethal H1N1 influenza A infection is attenuated by recombinant murine activated protein C treatment**. Levels of **A. and C**. thrombin-antithrombin complexes (TATc) and **B. and D**. fibrin degradation products (FDP) in A. and B. lung and C. and D. plasma at baseline (dashed) and 48 and 96 hours after induction of lethal influenza A infection in buffer control treated mice (white) and recombinant murine activated protein C treated mice (grey). Data are expressed as box-and-whisker diagrams depicting the smallest observation, lower quartile, median, upper quartile and largest observation (eight mice per group at each time point). ^## ^and ^### ^indicate statistical significance as compared to baseline (*P *< 0.01 and *P *< 0.001 respectively, Mann-Whitney U test), ** and *** indicate statistical significance as compared to buffer control (*P *< 0.01 and *P *< 0.001 respectively, Mann-Whitney U test).

Administration of APC has been found to inhibit activation of coagulation in animals and patients with severe bacterial sepsis [13,16,28]. To determine the effect of APC on the procoagulant response in severe influenza, we infected mice with a lethal dose of influenza A virus and initiated rm-APC (or buffer control) treatment 24 hours after infection; subsequently, we determined the levels of TATc and FDP in lung homogenates (Figure 1A, B) and plasma (Figure 1C, D) at 48 and 96 hours after infection. Inoculation with a lethal dose of influenza significantly increased the levels of TATc and FDP in both lung homogenates and plasma after 48 and 96 hours as compared to uninfected control mice. Treatment with rm-APC strongly inhibited local and systemic activation of coagulation as shown by decreased levels of TATc and FDP in rm-APC treated animals in both lungs and plasma (Figure 1A-D).

**Figure 2:**
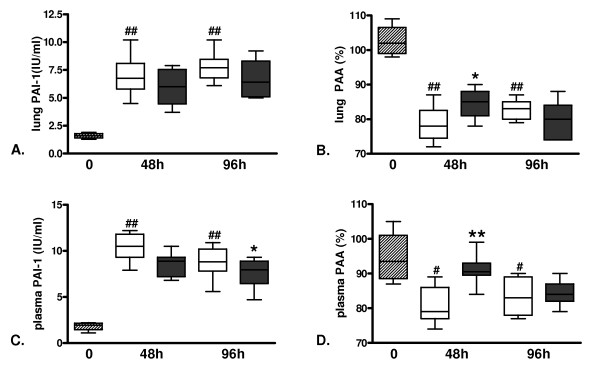
**Induction of plasminogen activator inhibitor type-1 and downregulation of fibrinolysis in lethal H1N1 influenza A infection is partially reversed by recombinant murine activated protein C treatment**. Levels of **A. and C**. plasminogen activator inhibitor type-1 (PAI-1) and **B. and D**. plasminogen activator activity (PAA) in A. and B. lung and C. and D. plasma at baseline (dashed) and 48 and 96 hours after induction of lethal influenza A infection in buffer control treated mice (white) and recombinant murine activated protein C treated mice (grey). Data are expressed as box-and-whisker diagrams depicting the smallest observation, lower quartile, median, upper quartile and largest observation (eight mice per group at each time point). ^# ^and ^## ^indicate statistical significance as compared to baseline (*P *< 0.05 and *P *< 0.01 respectively, Mann-Whitney U test), * and ** indicate statistical significance as compared to buffer control (*P *< 0.05 and *P *< 0.01 respectively, Mann-Whitney U test).

Evidence derived from *in vitro *investigations indicates that APC may stimulate fibrinolysis by inhibiting the main inhibitor of this system, PAI-1 [29,30]. To study the effect of lethal influenza A infection on fibrinolysis and the impact of rm-APC treatment hereon, we determined the levels of PAI-1 and PAA in lung homogenates and plasma. In influenza PAI-1 levels were increased at 48 and 96 hours both locally and systemically as compared to controls (Figure 2A, C), which was associated with downregulation of PAA in both lung and plasma (Figure 2B, D). Treatment with rm-APC reduced PAI-1 concentrations in lung and plasma, but this was only significant in plasma 96 hours after infection. Although the effect of rm-APC on PAI-1 was not significant at 48 hours, rm-APC treatment did partially preserve fibrinolytic activity at 48 hours both in lung and plasma, as indicated by higher PAA as compared to buffer control treated mice (Figure 2B, D). After 96 hours differences in PAA had subsided.

Of note, no bleeding complications were seen in mice treated with rm-APC, except for the occasional small peritoneal haematomas at the injection site, which were not seen in buffer control treated mice.

### Lung inflammation

**Figure 3:**
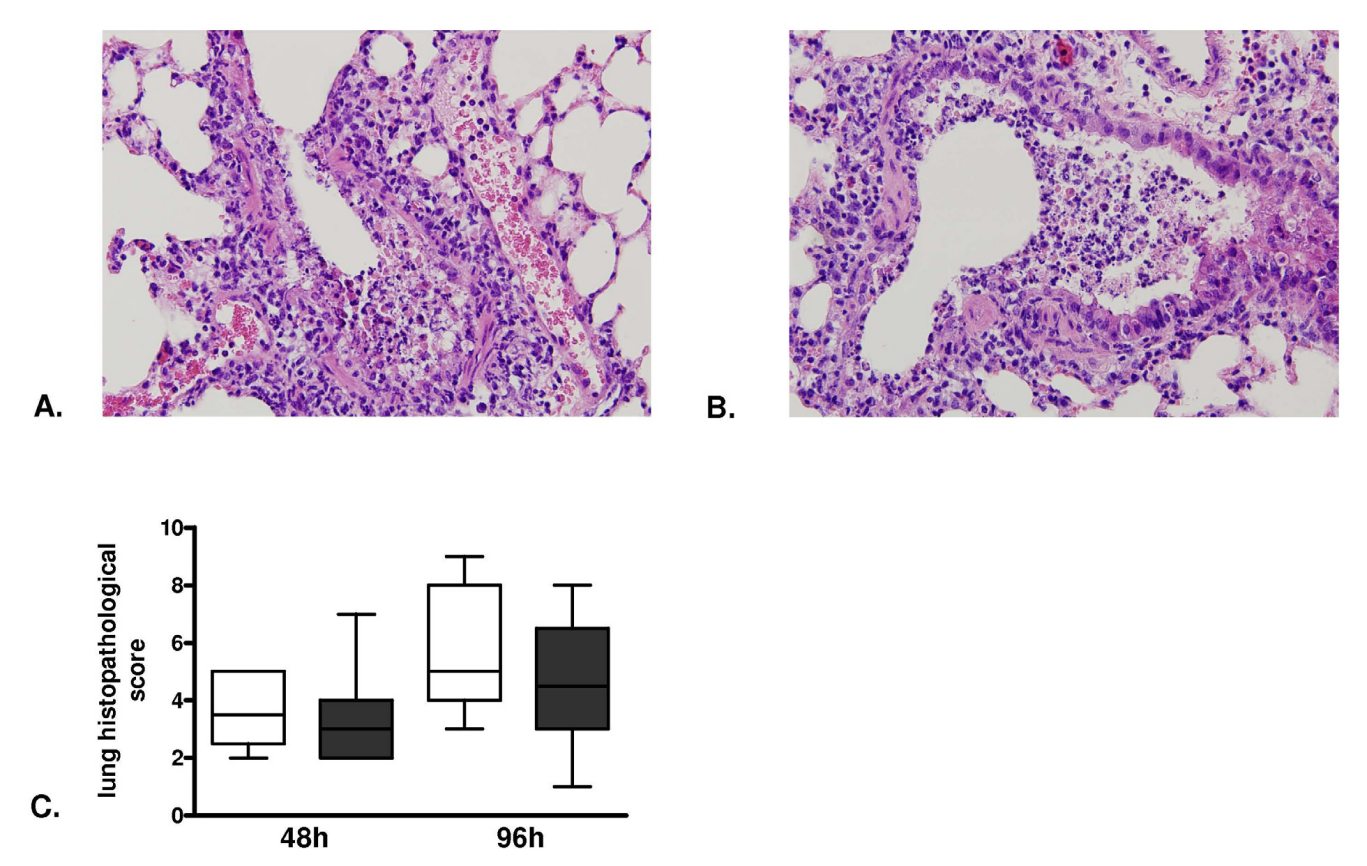
**Lung histopathology in lethal H1N1 influenza A infection is not influenced by recombinant murine activated protein C treatment**. Representative slides of lung haematoxylin and eosin staining 96 hours after induction of lethal influenza A infection in **A**. buffer control treated mice and **B**. recombinant murine activated protein C treated mice (original magnification × 100). **C**. Total pathology score (described in methods section) 48 and 96 hours after induction of lethal influenza A infection in buffer control treated mice (white) and recombinant murine activated protein C treated mice (grey). Data are expressed as box-and-whisker diagrams depicting the smallest observation, lower quartile, median, upper quartile and largest observation (eight mice per group at each time point). No statistical differences between the groups at each time point.

Lethal influenza was associated with pulmonary inflammation and damage as evidenced by the occurrence of bronchitis, interstitial inflammation, oedema and endothelialitis both at 48 hours (pictures not shown) and 96 hours after infection (Figure 3A, B). There were no differences in total histopathological scores between rm-APC and buffer control treated mice at either 48 or 96 hours after infection (Figure 3C). Moreover, there were no differences in the separate scores for bronchitis, interstitial inflammation, oedema and endothelialitis (not shown).

**Figure 4:**
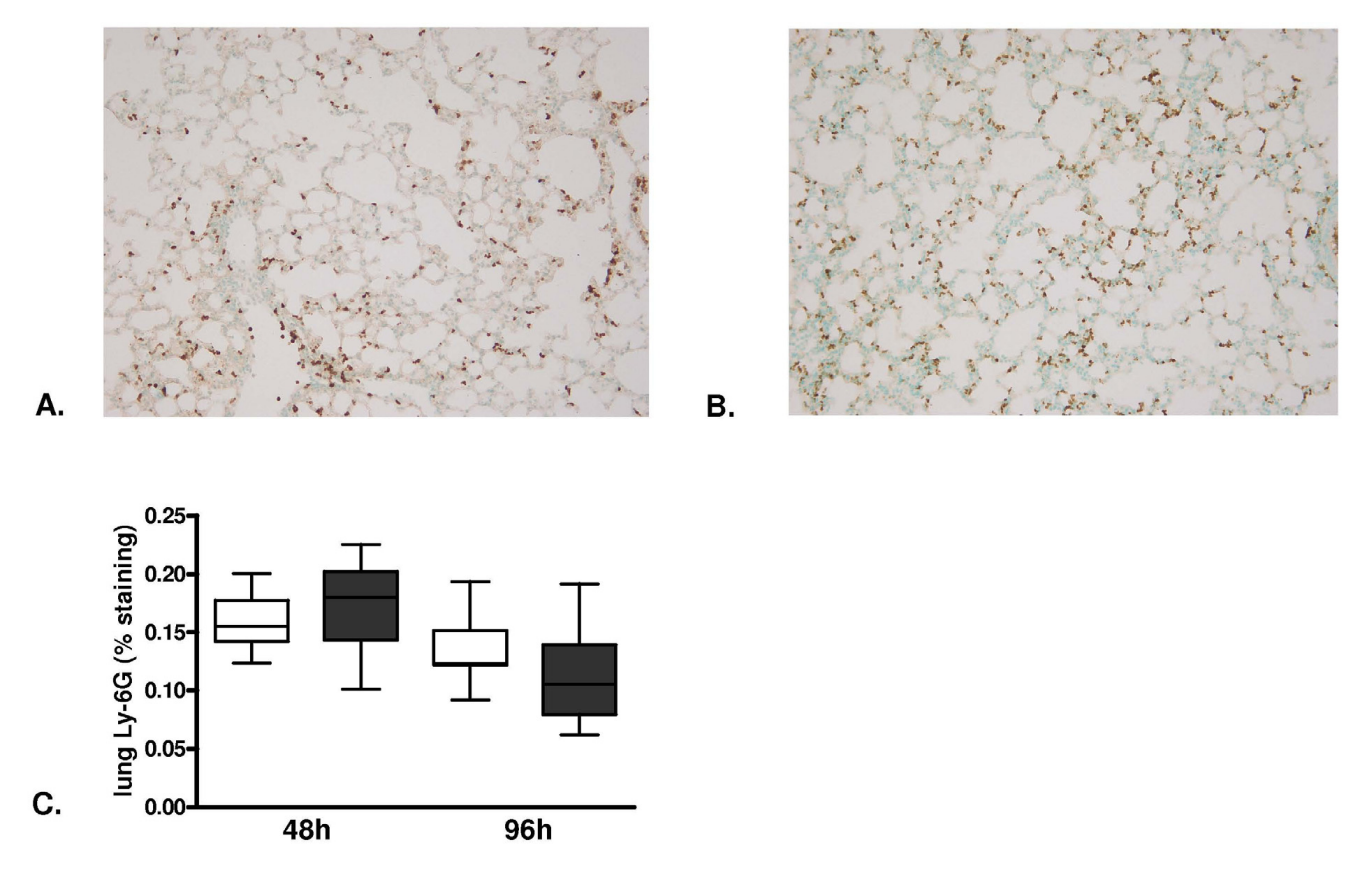
**Lung neutrophil influx in lethal H1N1 influenza A infection is not influenced by recombinant murine activated protein C treatment**. Representative slides of lung Ly-6G staining (brown) 96 hours after induction of lethal influenza A infection in **A**. buffer control treated mice and **B**. recombinant murine activated protein C treated mice (original magnification × 100). **C**. Quantitation of pulmonary Ly-6G content 48 and 96 hours after induction of lethal influenza A infection in buffer control treated mice (white) and recombinant murine activated protein C treated mice (grey). Data are expressed as box-and-whisker diagrams depicting the smallest observation, lower quartile, median, upper quartile and largest observation (eight mice per group at each time point). No statistical differences between the groups at each time point.

One of the prominent features in lethal influenza is neutrophil influx into the lung parenchyma both after 48 hours (pictures not shown) and 96 hours (Figure 4A, B). There were no differences in neutrophil influx between rm-APC and buffer control treated mice after 48 or 96 hours, as evidenced by equal percentages of positivity in Ly-6G stainings (Figure 4C). In line, pulmonary MPO concentrations, indicative for the number of neutrophils in lung tissue, were similar in both treatment groups at both time points (Table 1).

**Table 1:** Pulmonary myeloperoxidase, cytokine and chemokine levels 48 and 96 hours after induction of lethal H1N1 influenza A infection

	Placebo	rm-APC	placebo	rm-APC
**MPO (ng/ml)**	5.0 (3.9 to 5.9)	4.9 (4.2 to 5.1)	6.4 (5.6 to 6.9)	6.8 (6.2 to 8.9)
**TNF-α (pg/ml)**	120 (97 to 134)	121 (101 to 143)	393 (293 to 495)	167 (139 to 344) *
**IL-1β (pg/ml)**	236 (100 to 415)	389 (331 to 389)	479 (444 to 549)	371 (345 to 587)
**IL-6 (ng/ml)**	0.9 (0.7 to 1.0)	0.8 (0.7 to 0.9)	1.3 (1.0 to 1.6)	1.0 (0.7 to 1.3)
**IL-12 (pg/ml)**	B.D.	B.D.	54 (37 to 64)	11 (5.0 to 28) *
**IL-10 (pg/ml)**	137 (86 to 165)	131 (101 to 153)	115 (82 to 175)	65 (23 to 147)
**IFN-γ (pg/ml)**	6.8 (6.0 to 9.7)	6.9 (5.8 to 7.9)	6.7 (4.9 to 8.1)	5.6 (3.5 to 7.9)
**KC (ng/ml)**	3.1 (2.3 to 3.7)	4.0 (2.9 to 5.1)	3.4 (2.6 to 4.4)	4.2 (3.3 to 5.6)
**MIP-2 (ng/ml)**	1.2 (0.8 to 1.4)	1.5 (1.2 to 1.6)	1.4 (1.3 to 1.5)	1.5 (1.4 to 1.7)

Data are medians (interquartile ranges) of eight mice per group at each time point. * indicates statistical significance compared to placebo (*P *< 0.05). MPO, myeloperoxidase, TNF-α, tumor necrosis factor-α, IL, interleukin, IFN-γ, interferon-γ, KC, keratinocyte-derived chemokine, MIP-2, macrophage inflammatory protein-2, B.D., below detection.

To further investigate the effect of rm-APC treatment on the inflammatory response in severe influenza, we determined pulmonary levels of various cytokines (TNF-α, IL-1β, IL-6, IL-10, IL-12p70, IFN-γ) and chemokines (KC, MIP-2) in lung homogenates obtained 48 and 96 hours after infection. After 48 hours of infection, there were no statistically significant differences in cytokine levels between rm-APC and buffer control treated animals (Table 1). After 96 hours, the levels of the pro-inflammatory cytokines TNF-α and IL-12p70 were lower in rm-APC treated animals. Levels of KC and MIP-2 did not differ between treatment groups at any time point.

### Viral load

**Figure 5:**
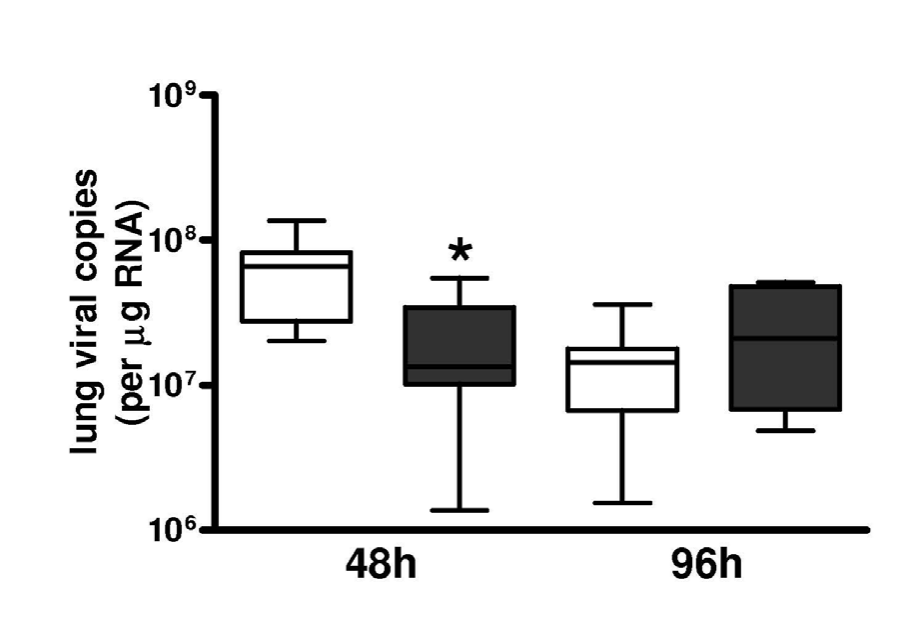
**Pulmonary viral loads in lethal H1N1 influenza A infection are transiently reduced by recombinant murine activated protein C treatment**. Lung viral RNA copies 48 and 96 hours after induction of lethal influenza A infection in buffer control treated mice (white) and recombinant murine activated protein C treated mice (grey). Data are expressed as box-and-whisker diagrams depicting the smallest observation, lower quartile, median, upper quartile and largest observation (eight mice per group at each time point). * indicates statistical significance as compared to buffer control (*P *< 0.05, Mann-Whitney U test).

To investigate the effect of rm-APC on the antiviral response in influenza infection, we determined viral loads in lungs over time. Remarkably, after 48 hours, rm-APC treatment was associated with more than four-fold less viral RNA copies as compared to buffer control treatment (Figure 5). However, after 96 hours after infection this difference in viral load had subsided completely.

### Survival

**Figure 6:**
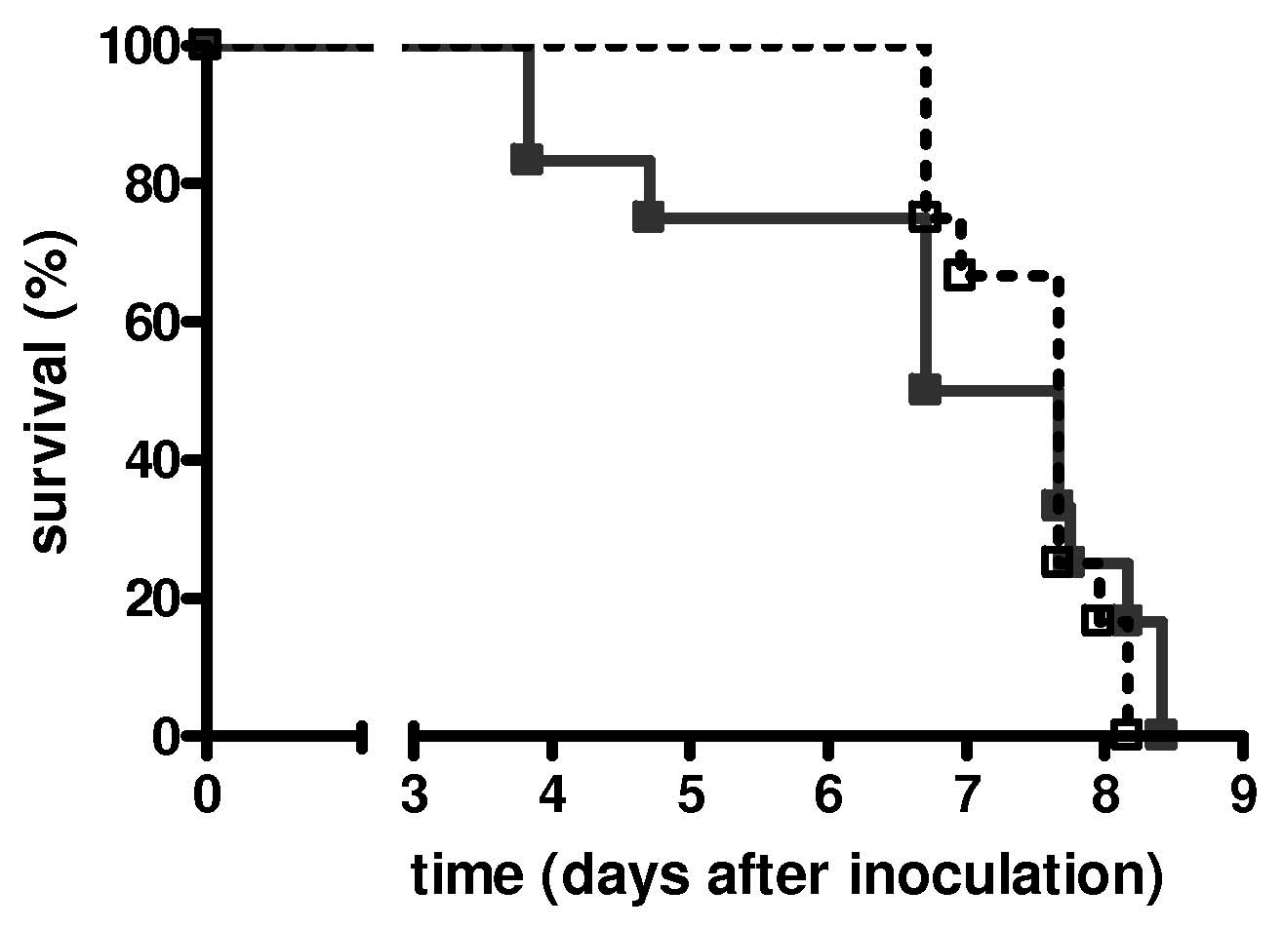
**Survival in lethal H1N1 influenza A infection is not affected by recombinant murine activated protein C treatment**. Survival of buffer control treated mice (open squares, n = 12) and recombinant murine activated protein C treated mice (grey squares, n = 12) in lethal influenza A infection. No statistical differences between the groups (log rank test).

To substantiate whether differences in activation of coagulation, downregulation of fibrinolysis, viral loads and TNF-α and IL-12p70 levels between rm-APC and buffer control treated animals were associated with an altered mortality we performed a survival study. The infection was associated with 100% lethality within nine days in both treatment groups and mortality curves did not differ between rm-APC and buffer control treated mice (Figure 6).

## Discussion

Influenza is an important cause of pneumonia, causing 5 to 10% of all CAP cases [4,7]. While bacterial pneumonia has been linked to activation of coagulation and downregulation of anticoagulant mechanisms and fibrinolysis [10-12], knowledge of the impact of influenza on hemostasis is limited. We here studied alterations in local and systemic activation of coagulation and fibrinolysis together with induction of inflammation during lethal influenza A infection. In addition, considering that previous investigations in patients and animals have especially pointed to beneficial effects of APC treatment in the lungs [17,31-33], we determined the effect of APC on the procoagulant and inflammatory response to and the outcome of lethal influenza. We show that lethal H1N1 influenza A infection is associated with extensive pulmonary and systemic activation of coagulation accompanied by inhibition of fibrinolysis. Systemic administration of APC, started 24 hours after infection, mimicking a possible clinical scenario, strongly attenuated coagulation activation and partially reversed inhibition of fibrinolysis, but did not influence lung inflammation or survival.

Concurrent alterations in coagulation and fibrinolysis during influenza have not been studied in detail thus far. One clinical study in children has indicated that severe influenza can be associated with disseminated intravascular coagulation [18]. In addition, mice with non-lethal influenza A infection displayed a rise in plasma TATc and PAI-1 levels; although fibrinolytic activity (such as measured by PAA) was not determined in this previous investigation, these data point to concurrent activation of coagulation and inhibition of fibrinolysis at the systemic level during mild influenza [19]. Our own preliminary data have suggested that lethal influenza not only results in systemic coagulation activation, but also in induction of the coagulation system in the lungs (Schouten et al, XXIst Congress of the International Society of Thrombosis and Haemostasis, Boston, July 2009, abstract no. 3065). Our current results confirm and expand these previous data. First, we demonstrate local and systemic activation of coagulation, as evidenced by increased lung and plasma TATc and FDP levels in influenza infected mice at 48 and 96 hours. Moreover, we show that activation of coagulation is accompanied by local as well as systemic downregulation of fibrinolysis, as reflected by elevated PAI-1 and reduced PAA levels in lung homogenates and plasma, which probably further contributes to the influenza-induced procoagulant state. Most likely, the downregulation of fibrinolytic activity can be explained at least partially by upregulation of PAI-1, the main inhibitor of the fibrinolytic system. As such, severe influenza appears to cause similarly opposite changes in pulmonary coagulation and fibrinolysis as previously reported for bacterial pneumonia and acute respiratory distress syndrome [34-37].

Systemic administration of rm-APC strongly inhibited activation of the coagulation system, as indicated by markedly reduced plasma and lung concentrations of TATc and FDPs in rm-APC treated mice relative to vehicle treated animals. In addition, rm-APC had a modest but statistically significant effect on the fibrinolytic system, partially blunting the influenza-induced rise in plasma and lung PAI-1 levels and partially preserving plasma and lung fibrinolytic activity. The capacity of APC to attenuate systemic coagulation during severe bacterial infection has been demonstrated in several studies [13,16,38]. Our group previously reported on the effects of intravenous administration of recombinant APC on pulmonary coagulation in healthy humans intrabronchially challanged with lipopolysaccharide (LPS) [39] and in rats challenged with LPS systemically [40] or with viable bacteria via the airways [41,42]. All of these previous studies [39-42], in which APC treatment was started before the challenge with LPS or bacteria, revealed the capacity of APC to inhibit coagulation in the lungs. The current study adds to these earlier findings that APC is capable of inhibiting systemic and local coagulation during influenza-induced pneumonia and that this effect is present when APC administration is initiated 24 hours after infection, that is, in a clinically more relevant setting. Of interest, endogenous APC may also reduce influenza-induced coagulation, as indicated by studies in mice with a mutation in their *thrombomodulin *gene that results in a minimal capacity for endogenous APC generation: these mice demonstrated increased plasma levels of TATc (relative to wild-type mice) during non-lethal influenza [19]. Our finding that rm-APC stimulated fibrinolysis by inhibiting PAI-1 is supported by evidence derived from *in vitro *investigations [29,30]. Of note, previous studies from our laboratory could not demonstrate an effect of recombinant APC on pulmonary fibrinolysis during LPS-induced lung injury [39,40] or bacterial pneumonia [41,42].

Besides anticoagulant and profibrinolytic properties, APC has been found to exert anti-inflammatory activity (reviewed in [13]). Previous studies have suggested that recombinant APC can inhibit LPS-induced neutrophil recruitment and activation in the lungs [31,43]. Nonetheless, in the current study rm-APC did not have a major impact on lung inflammation during lethal influenza A infection, as indicated by similar histopathology scores of lung tissue, a similar influx of neutrophils to the site of infection and largely similar cytokine and chemokine concentrations in lung homogenates. Interestingly, rm-APC did reduce lung TNF-α and IL-12 levels 96 hours after infection; similarly, APC has been found to inhibit the LPS-induced production of TNF-α *in vitro *and *in vivo *[32,44].

To our knowledge, the effect of APC on antiviral defense per se has not been studied. We here show that rm-APC temporarily lowers pulmonary viral loads about four-fold, as measured 48 hours after infection. These differences between rm-APC and vehicle treated mice had disappeared 96 hours post infection. The transiently reduced viral loads in rm-APC treated animals are surprising considering that APC is not known to impact on antiviral mechanisms and did not influence the inflammatory response to influenza A in a way that might have improved host defense. The difference in viral load between rm-APC and buffer treated mice did not result in a substantially changed inflammatory response or a delayed mortality. However, since we tested only one infectious dose of influenza A, we cannot exclude that rm-APC does impact on lethality after infection with different viral doses. The mechanism by which rm-APC reduces viral loads at an early stage of influenza infection needs further investigation. Besides anticoagulant and anti-inflammatory properties, APC has been described to influence the hemodynamic response to an inflammatory stimulus [13,45]. The potential effect of rm-APC on hemodynamics was not measured in our current study and therefore warrants further investigation.

In order to mimic the clinical situation, APC should be administered by a continuous intravenous infusion. However, this is difficult to achieve in mice for a period of several days. In this study, we therefore administered rm-APC intraperitoneally every eight hours at a dose of 125 μg (a daily dose of approximately 15 mg/kg, that is, approximately 25 times higher than the daily dose administered to humans). This administration protocol resulted in plasma levels which were not dissimilar to the levels observed after intravenous administration of lower doses in previous studies in rodents in which anti-inflammatory effects of recombinant APC were demonstrated after LPS administration [31,32,46,47] and which are in the same range as those achieved by continuous intravenous infusion in septic patients [48]. In light of these earlier rodent and patient investigations [31,32,46-48] and considering that the APC dosing schedule used here caused profound anticoagulant effects, we consider it unlikely that higher APC doses would have had a significant effect on lung inflammation or survival. Such studies would be less clinically relevant and moreover would be associated with an increased risk for bleeding, which was not observed with the current dosing regimen. It would be of considerable interest, however, to study the effects of mutant forms of APC with reduced anticoagulant but enhanced cytoprotective properties in models of lethal influenza [46,47,49].

## Conclusions

Lethal H1N1 influenza infection is associated with both pulmonary and systemic activation of coagulation and inhibition of fibrinolysis. Rm-APC treatment, started 24 hours after the onset of infection, partially prevents these hemostatic derangements, but does not impact on lung inflammation or survival.

## Key messages

• Lethal H1N1 influenza infection is associated with both pulmonary and systemic activation of coagulation and inhibition of fibrinolysis.

• Rm-APC treatment, started 24 hours after the onset of lethal H1N1 infection, partially prevents influenza-induced procoagulant and anti-fibrinolytic derangements.

• Rm-APC treatment, started 24 hours after the onset of lethal H1N1 infection, does not impact on lung inflammation or survival.

## Abbreviations

APC: activated protein C; CAP: community-acquired pneumonia; CBA: cytometric bead array; FDP: fibrin degradation products; IFN-γ: interferon-γ; IL: interleukin; LPS: lipopolysaccharide; KC: keratinocyte-derived chemokine; MIP-2: macrophage inflammatory protein-2; MPO: myeloperoxidase; PAA: plasminogen activator activity; PAI-1: plasminogen activator inhibitor-1; RAGE: receptor for advanced glycation end products; rh-/rm-: recombinant human/mouse; TATc: thrombin-antithrombin complexes; TNF-α: tumor necrosis factor-α.

## Competing interests

Bruce Gerlitz and Brian Grinnell are employed by Lilly Research Laboratories, a division of Eli Lilly & Co, which produces recombinant human APC for the treatment of severe sepsis. The other authors declare they have no conflicts of interests.

## Authors' contributions

MS participated in the design of the study, carried out the *in vivo *experiments and drafted the manuscript. KFS participated in the design of the study and helped to draft the manuscript. BG and BWG provided the rm-APC and participated in the design of the study. JJTHR performed pathology scoring, prepared part of the figures and helped to draft the manuscript. ML performed coagulation measurements and helped to draft the manuscript. CV participated in the design of the study, advised in laboratory matters and helped to draft the manuscript. TP participated in the design of the study, supervised the study and helped to draft the manuscript. All authors read and approved the manuscript.
